# Morphometric characterization and decision tree–based prediction of phenotypic traits in Pantaneiro sheep

**DOI:** 10.1007/s11250-026-05088-5

**Published:** 2026-05-28

**Authors:** Agda Costa Valério, Tatiane Fernandes, Adrielly Lais Alves da Silva, Renata Alves Chagas, Ariadne Patrícia Leonardo, Marcio Rodrigues de Souza, Núbia Michelle Vieira da Silva, Fernando Miranda de Vargas Junior

**Affiliations:** 1 Faculty of Agrarian Sciences, Federal University of Grande Dourados, Dourados, MS Brazil; 2 Virginia Tech, School of Animal Sciences, Blacksburg, USA; 3 Federal Institute of Mato Grosso do Sul, Campus Dourados, Dourados, MS Brazil

**Keywords:** Body condition score, Biometric traits, Multivariate analysis, Decision tree, Conservation

## Abstract

The Pantaneiro sheep is a locally adapted genetic resource shaped by the seasonal flooding, thermal stress, and heterogeneous forage availability of the Brazilian Pantanal. Characterizing its morphological structure using objective and field-applicable traits is essential for standardized phenotyping and conservation-oriented breeding strategies. This study evaluated 211 Pantaneiro ewes raised under semi-extensive management to identify morphometric patterns and identify simple predictors of qualitative phenotypic traits. Twenty-three quantitative and ten qualitative traits were measured and analyzed using multivariate techniques, including factor analysis and hierarchical clustering, to describe morphological structure. Decision tree models were then developed to predict key phenotypic traits from morphometric variables. Four main morphological dimensions explained 64.6% of total variance, reflecting body capacity, limb robustness, and cranial morphology. Among the ten decision tree models generated, three achieved predictive accuracies above 80% head profile (86.3%), muzzle type (84.8%), and wool color (82.5%), indicating that metatarsus and tarsus circumferences are reliable non-invasive predictors of cranial and tegumentary traits. The integration of multivariate morphometry and machine learning proved effective for identifying simple predictors applicable to field management. Overall, these findings reinforce the adaptive potential of Pantaneiro ewe and their importance for sustainable and resilient livestock production in tropical floodplain ecosystems.

**Supplementary Information:**

The online version contains supplementary material available at 10.1007/s11250-026-05088-5.

## Graphical Abstract

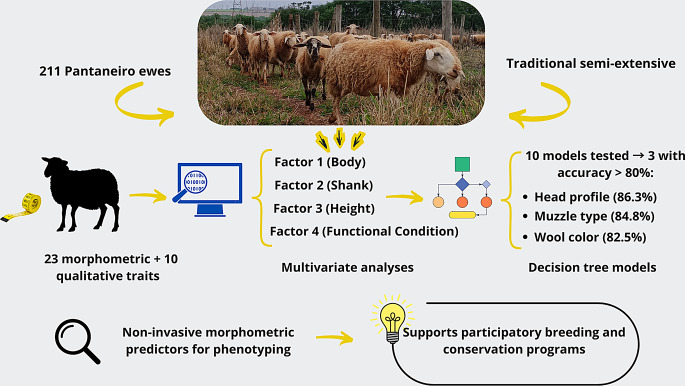

## Introduction

The Pantanal, recognized as a Brazilian National Heritage site in 1988 and designated a UNESCO Biosphere Reserve in 2000 (Padovani 2017), is one of the world’s largest floodplains, covering approximately 150,988 km², or 1.8% of the Brazilian territory (IBGE, 2019). Classified as a tropical savanna climate (Aw) according to the Köppen-Geiger climate classification (Beck et al. 2018), originally proposed by Köppen (1948), the region is strongly shaped by a seasonal hydrological regime that imposes intense selective pressures on local fauna and livestock production systems.

Within this ecological framework, Pantaneiro sheep emerged as a native Brazilian genetic resource, whose hardiness results from centuries of natural selection under the harsh edaphoclimatic conditions of the Pantanal, a seasonal ecosystem characterized by recurrent flooding (Monteschio et al. 2018; Sousa et al. 2024). These animals exhibit distinctive morpho-functional traits that support their adaptive capacity and productive potential under low-input systems (Crispim et al. 2017; Vargas Junior et al. 2019). Despite their ecological and genetic relevance, Pantaneiro sheep remain undercharacterized when compared with commercial breeds, particularly with respect to quantitative phenotypic structure.

Biometric characterization provides an essential tool to understand genetic diversity, adaptive attributes, and productive performance in small ruminants (Oliveira et al. 2020; Sousa et al. 2024). Morphometric studies, based on measurements of external body dimensions, are widely used to identify breed traits, assess intra- and inter-group variability, and support conservation and breeding programs (Bakhshalizadeh et al. 2016; Brito et al. 2021). Furthermore, biometric traits have the potential to predict carcass characteristics and overall productivity, expanding their use as a management and selection tool (Gomes et al. 2021; Gurgel et al. 2021).

Key biometric indicators, such as withers height, body length, thoracic perimeter, and limb circumference, are often correlated with body weight and carcass composition, allowing indirect selection for economically relevant traits (Bakhshalizadeh et al. 2016; Chay-Canul et al. 2024). When combined with advanced statistical tools, biometric data can reveal complex phenotypic relationships beyond linear associations. Recent studies have demonstrated that multivariate approaches and machine learning-basead models ares effective in reducing data dimensionality, identifying trait clusters, and predicting performance-related parameters, offering practical opportunities for optimizing selection and management in native breeds, particularly in data-limited tropical systems.

Body condition score (BCS) is a widely used and biologically meaningful indicator in small ruminants, as it reflects body fat reserves, nutritional status, and overall energy balance. It is strongly associated with productive performance, reproductive efficiency, health status, and adaptive capacity, particularly under extensive and semi-extensive tropical production systems. In this context, the present study aimed to investigate the relationship between biometric measurements and body condition score (BCS) in Pantaneiro ewes, employing multivariate statistics and decision tree models to identify phenotypic markers associated with body condition. Understanding how body conformation relates to BCS can improve selection accuracy, inform feeding strategies, and strengthen conservation-oriented breeding programs adapted to tropical production environments.

## Materials and methods

All experimental procedures were approved by the Animal Experimentation Ethics Committee (CEUA) of the Federal University of Grande Dourados (UFGD), Dourados, Mato Grosso do Sul State, Brazil.

### Environmental setting and sheep population

The study was conducted in the microregion of Dourados, Mato Grosso do Sul, Brazil, a transitional zone between the Pantanal biome and the Cerrado savanna, representing an ecotonal area of high biodiversity. The experimental site is located between 22°13′S and 22°18′S latitude and 54°48′W and 55°05′W longitude, with an average altitude of 430–460 m above sea level. The climate is tropical with alternating wet and dry seasons ((Aw according to Köppen-Geiger classification; (Köppen 1948). Annual rainfall averages 1,400 mm, concentrated between October and March. The soil is predominantly clayey, and the topography is gently undulating.

A total of 211 Pantaneiro ewes were sampled from the Sheep Research Center of the Faculty of Agricultural Sciences, Federal University of Grande Dourados (UFGD), located in Dourados, MS. The flock was managed under a traditional semi-extensive system characterized by low technification and minimal infrastructure. During daylight hours, animals grazed on pastures predominantly composed of *Cynodon* spp. (Tifton) and *Urochloa decumbens* (signal grass). At night, the sheep remained outdoors, with little or no shelter provided. Nutritional supplementation consisted solely of mineral salt, offered *ad libitum*.

### Quantitative and qualitative morphometric measurements

Quantitative morphometric measurements were taken using a measuring tape and a zoometric stick, always by the same trained evaluator, to minimize inter-observer variation. The methodology was adapted from Oliveira et al. (2014). The following 23 variables were recorded: head length, skull length, head width, face length, ear length, neck girth, neck length, body length, body depth, shoulder width, chest girth, croup length, width between ilia, width between ischia, withers height, crop height, height from ground to belly, tarsus girth, metatarsus girth, carpus girth, metacarpus girth, foreleg length, and hindleg length.

**Fig. 1:**
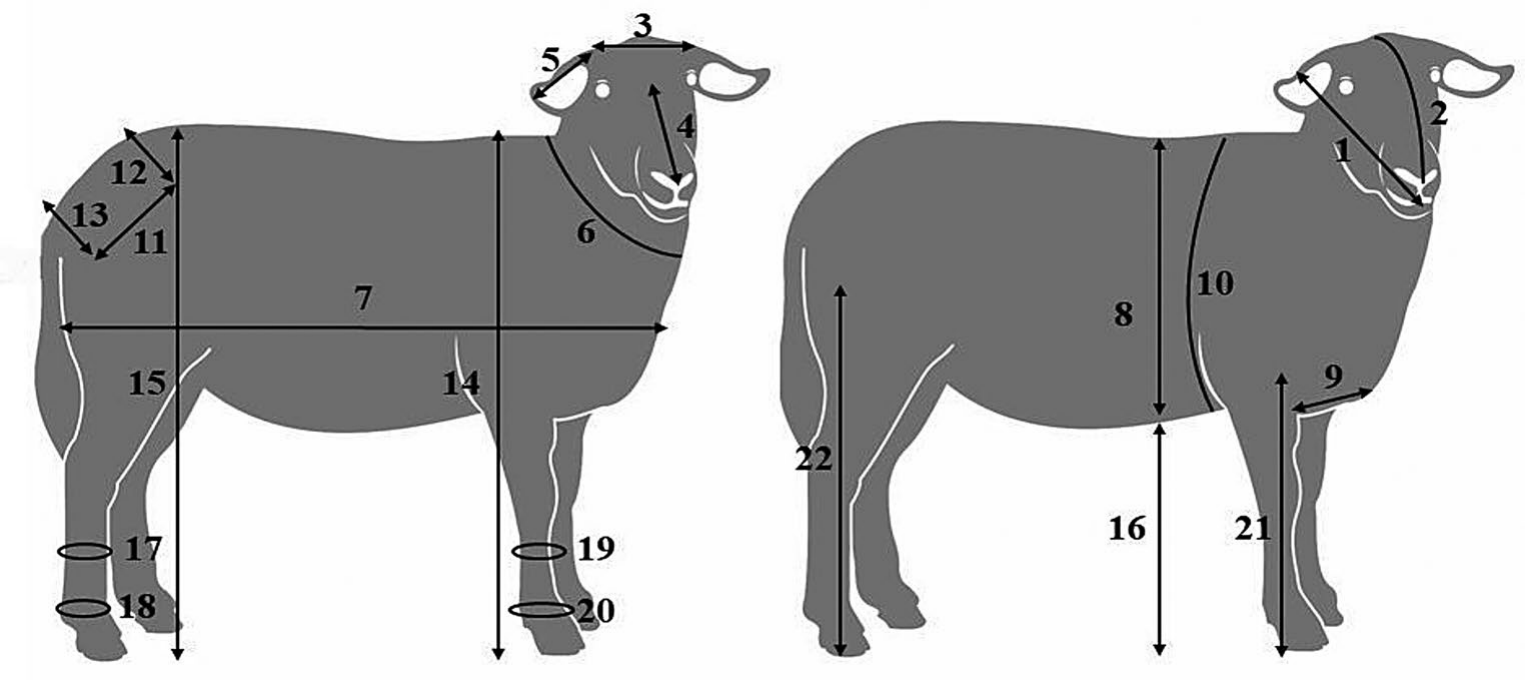
Illustration of the quantitative morphological measurements analyzed in Pantaneira ewes. 1 = Head length, 2 = Skull length, 3 = Head width, 4 = Face length, 5 = Ear lenght, 6 = Neck girth, 7 = Body length, 8 = Body depth, 9 = Shoulder width, 10 = Chest girth, 11 = Croup length, 12 = Iliac width, 13 = Ischial width, 14 = Withers height, 15 = Crop height, 16 = Ground-belly distance, 17 = Tarsus girth, 18 = Metatarsus girth, 19 = Carpus girth, 20 = Metacarpus girth, 21 = Foreleg length and 22 = Hindleg lenght. Figure adapted from Aranda (2021)

In total, 23 variables were studied. Figure 1 illustrates 22 of these measurements. Neck length, although evaluated, was not represented graphically because its anatomical landmarks are not clearly defined in a two-dimensional lateral schematic.

Qualitative morphological characterization was performed based on visual scores developed by the authors, evaluating: head profile (concave, convex, or straight), nasal bridge (concave, convex, or straight), muzzle (narrow or broad), horns (absent, scurred, or normal), markings (belly, head, ears, and/or legs), eye rings (‘glasses’; absent, bilateral, or unilateral), coat color (yellow, white, spotted, or black), wool color (yellow, white, spotted, or black), skin color (white, depigmented, dark, or spotted), and hooves (white, mixed, black, or striped) (Aranda et al. 2021).

### Statistical analysis

Descriptive and multivariate statistical analyses were performed using MINITAB^®^ software (version 17, Minitab LLC, State College, PA, USA) and the R environment (version 4.3.3; R Core Team, Vienna, Austria). Data suitability for factor analysis was assessed using the Kaiser–Meyer–Olkin (KMO) measure and Bartlett’s test of sphericity. Quantitative morphological variables were then subjected to exploratory factor analysis to reduce data dimensionality and identify latent structures.

Based on the factor structure obtained, hierarchical clustering was performed to define training subsets, which were then used for constructing decision tree-based prediction models. Tree induction was guided by prior knowledge of the zootechnical relevance of the variables and the aim of obtaining parsimonious models that could generate informative splits without overfitting. Only models with predictive accuracy above 50% were considered adequate.

## Results

### Descriptive statistics

The descriptive statistics of the quantitative morphological measurements of Pantaneira ewes are presented in Table 1. Body condition score (BCS) and live weight exhibited the greatest variability (CV = 37.85% and 24.65%, respectively), reflecting heterogeneity primarily associated with nutritional status, but also influenced by age, physiological stage, health status, and individual metabolic efficiency within the flock. In contrast, height-related variables, such as withers height and crop height, displayed the lowest coefficients of variation (CV = 5.78% and 5.64%, respectively), suggesting stronger stabilizing selection for vertical skeletal development in this population. The mean live weight was 39.1 kg, while the average BCS was 1.5.

**Table 1:** Descriptive statistics for body condition score, live weight, and quantitative morphological measurements in Pantaneira ewes (*n* = 211)

Variable	Mean	SD	Min	Max	CV
Body condition score (1–5)	1.50	0.60	1.00	4.00	37.85
Live weight (kg)	39.09	9.64	20.00	62.00	24.65
Head length (cm)	26.13	1.84	21.00	31.00	7.05
Skull length (cm)	29.76	2.54	22.00	37.00	8.52
Head width (cm)	11.16	0.91	9.00	13.00	8.15
Face length (cm)	16.58	1.31	12.00	19.00	7.90
Ear length (cm)	15.35	1.55	11.00	19.00	10.09
Neck girth (cm)	33.32	3.50	25.00	46.00	10.51
Neck length (cm)	33.88	2.95	22.00	40.00	8.72
Body length (cm)	70.29	6.06	52.00	83.00	8.62
Body Depth (cm)	28.89	3.28	22.00	49.00	11.37
Shoulder width (cm)	19.03	1.91	14.00	24.00	10.03
Chest girth (cm)	80.19	6.77	65.00	96.00	8.44
Croup length (cm)	22.97	2.18	17.00	31.00	9.47
Iliac width (cm)	18.49	2.11	12.00	23.00	11.38
Ischial width (cm)	14.39	1.91	8.00	20.00	13.27
Withers height (cm)	63.78	3.68	53.00	75.00	5.78
Crop height (cm)	65.58	3.70	54.00	75.00	5.64
Ground-belly distance (cm)	36.87	2.88	28.00	46.00	7.81
Tarsus girth (cm)	9.08	0.70	7.00	10.00	7.74
Metatarsus girth (cm)	13.20	1.00	10.00	16.00	7.54
Carpus girth (cm)	7.77	0.61	6.00	10.00	7.80
Metacarpus girth (cm)	12.57	0.82	10.00	14.00	6.50
Foreleg length (cm)	45.54	4.54	26.00	57.00	9.98
Hindleg length (cm)	57.71	5.06	36.00	69.00	8.76

SD = standard deviation; min = minimum; max = maximum; CV = coefficient of variation

### Factor analysis

Factor loadings and communalities for the quantitative morphological traits of Pantaneira ewes are shown in Table 2. To facilitate interpretation, factor loadings with absolute values equal to or greater than 0.50 are highlighted. The four extracted factors explained 64.60% of the total variance, with individual contributions of 19.4%, 17.8%, 17.1%, and 10.3% for factors F1 through F4, respectively. Communalities ranged from 0.281 to 0.888, indicating an overall adequate representation of the original variables by the extracted factor structure.

Factor 1 (F1) was designated “Body”, as it was associated with high positive loadings for body depth, iliac width, chest girth, live weight, neck length, and ischial width—traits related to overall body volume, mass distribution, and structural capacity. Factor 2 (F2), termed “Shin”, grouped traits such as tarsus, metatarsus, carpal, and metacarpal girths, reflecting robustness of the distal limb segments and their role in structural support and locomotion. Factor 3 (F3), labeled “Height”, included high negative loadings for withers height, crop height, Ground-belly distance, and limb lengths (forelimb and hindlimb), highlighting vertical skeletal development. Factor 4 (F4), called “Functional Condition”, was primarily associated with body condition score, with additional contributions from shoulder width and live weight. This factor captures variation in the animals’ physiological and nutritional status, reflecting short-term functional condition rather than stable skeletal or conformational attributes.

These results reveal a consistent anatomical structure among Pantaneira ewes, supporting phenotypic modularity that may be useful for selection and conservation strategies based on multivariate profiles.

**Table 2:** Factor loadings and communalities for quantitative morphological traits of Pantaneira ewes

Variable	F1 Body	F2 Shank	F3 Height	F4 Functional Condition	Com
Body condition score	0.074	0.062	-0.067	**0.796**	0.648
Live weight	**0.617**	0.301	-0.401	**0.506**	0.888
Head length	0.081	**0.561**	-0.063	0.013	0.325
Face length	**0.571**	0.422	-0.292	-0.252	0.653
Ear length	0.133	0.445	-0.048	-0.250	0.281
Neck length	**0.617**	0.105	0.349	0.196	0.552
Body length	0.346	0.422	**-0.531**	0.337	0.693
Depth	**0.836**	0.082	-0.174	0.265	0.807
Shoulder width	0.449	0.097	-0.095	**0.686**	0.691
Chest girth	**0.639**	0.366	-0.362	0.423	0.852
Croup length	0.459	0.216	-0.373	0.310	0.493
Iliac width	**0.757**	0.365	-0.131	0.013	0.724
Ischial width	**0.541**	0.432	-0.227	0.047	0.533
Withers height	0.451	0.219	**-0.738**	0.124	0.812
Crop height	0.499	0.278	**-0.667**	0.133	0.789
Ground-belly distance	-0.040	-0.062	**-0.764**	-0.076	0.595
Tarsus girth	0.243	**0.782**	-0.058	0.062	0.678
Metatarsus girth	0.163	**0.723**	-0.250	0.136	0.631
Carpus girth	0.254	**0.676**	-0.086	0.319	0.631
Metacarpus girth	0.171	**0.719**	-0.194	0.260	0.651
Foreleg length	0.093	0.119	**-0.744**	0.207	0.619
Hindleg length	0.051	0.405	**-0.699**	0.016	0.655
**% Variance**	**19.40**	**17.80**	**17.10**	**10.30**	**64.60**

Com = communality

Highlighted values (in bold) indicate absolute loadings ≥ 0.50

### Decision tree models

A total of ten decision trees were generated, each corresponding to a qualitative trait. Three models achieved predictive accuracies above 80% and were retained in the main text due to their practical and zootechnical relevance: (i) head profile (86.3%), (ii) muzzle type (84.8%), and (iii) wool color (82.5%). These trees revealed consistent relationships between distal limb morphometrics and cranial or integumentary phenotypes in Pantaneiro ewes (Figs. 3 and 4).

In all decision tree diagrams, branches departing to the left represent a “yes” decision (criterion met), whereas branches departing to the right represent a “no” decision (criterion not met). This convention is displayed explicitly only in the root node by the software but applies consistently to all subsequent nodes.

The remaining models showed moderate to low accuracies (54.5–71.6%) and were included as Supplementary Figures (S1–S7). Although they contribute to the overall understanding of morpho-functional associations, their lower predictive performance or redundant patterns limit their application for selection purposes (Table 3).

**Table 3:** Decision tree models developed for predicting qualitative traits in Pantaneiro ewes, accuracy levels, and justification for inclusion in the main text or supplementary material

Target variable (qualitative trait)	Accuracy (%)	Justification	Location
Cephalic profile	86.25	Highest accuracy; morpho-functional relevance	Main text
Muzzle	84.83	High accuracy; practical relevance for selection	Main text
Wool color	82.46	Accuracy > 80%; non-obvious predictive link	Main text
Wool on head	65.87	Moderate accuracy; weaker limb association	Supplementary
Skin color	54.50	Low accuracy; limited predictive power	Supplementary
Ear spots	64.45	Intermediate accuracy; no added value	Supplementary
Head spots	67.29	Redundant with other markings	Supplementary
Belly Ear spots	71.56	Acceptable but no clear zootechnical impact	Supplementary
Leg Ear spots	70.14	Repetitive pattern with other markings	Supplementary
Glasses	68.24	Borderline accuracy; limited relevance	Supplementary

Decision tree analysis was employed to explore the relationships between quantitative morphometric variables and qualitative phenotypic traits in Pantaneiro ewes (Table 3). Trees were constructed using all variable groups identified in the hierarchical correlation analysis. Among them, the fourth group, consisting of distal limb segment measurements, yielded the best predictive performance, producing well-balanced tree structures.

For each qualitative trait, the algorithm automatically selected the variable forming the root node. Metatarsus grith was consistently chosen as the root predictor for several traits, including head profile, muzzle type, and wool color.

**Fig. 2:**
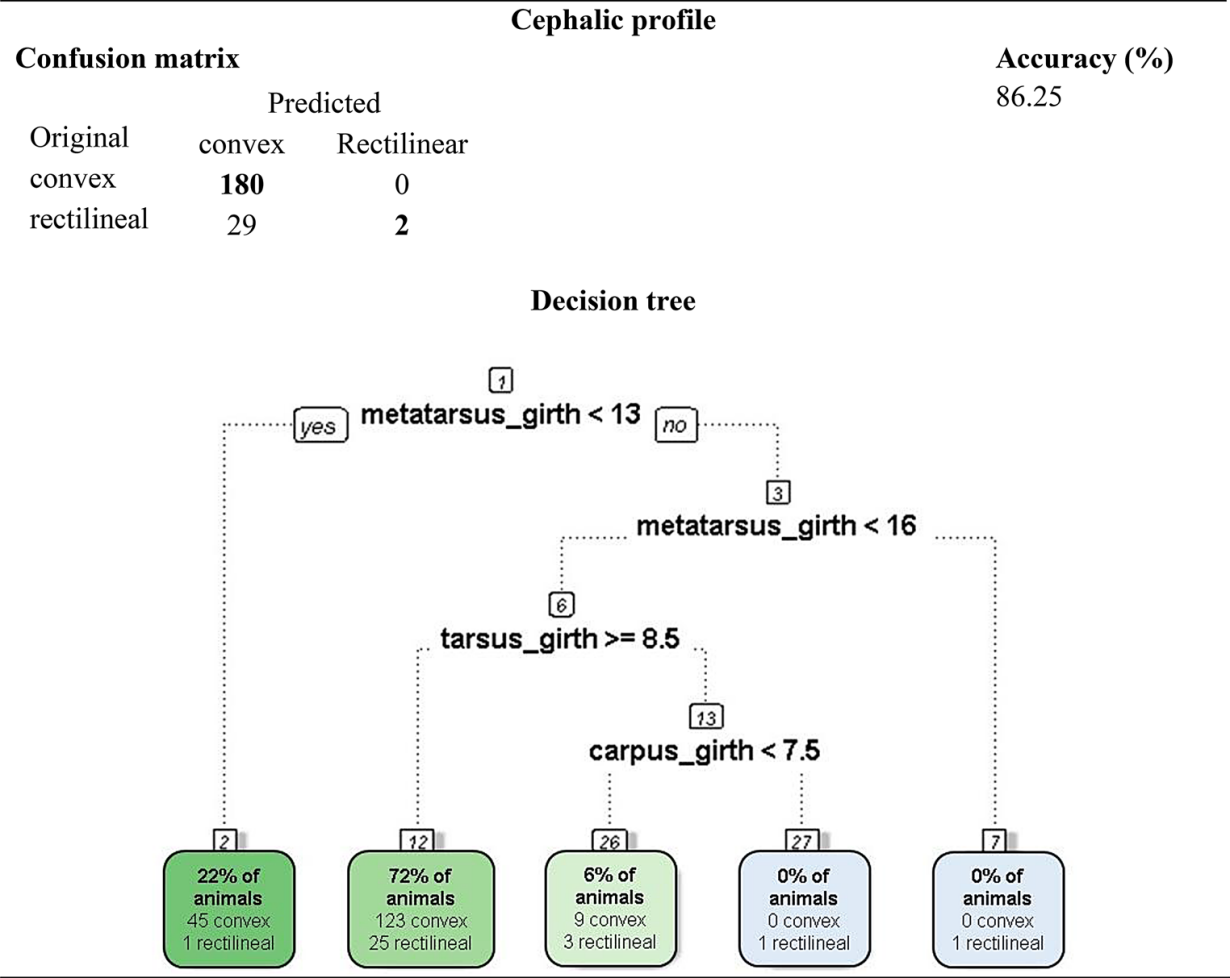
Confusion matrix and decision tree for predicting head profile type in Pantaneiro ewes based on limb morphometric measurements

Figure 2 illustrates the decision tree generated for predicting the head profile. In this model, metatarsus girth was selected as the root node. Ewes with metatarsus girth below 13 cm (22% of the sample) were almost exclusively classified as having a convex head profile, although one individual exhibited a straight profile. Among ewes with metatarsus girth between 13 and 16 cm and tarsus girth ≥ 8.5 cm, 72% were also classified as convex, despite 25 being straight. The corresponding confusion matrix indicated a predictive accuracy of 86.3%, highlighting the potential of distal limb measurements as non-invasive predictors of cranial phenotypic traits in Pantaneiro ewes.

The decision tree constructed for predicting muzzle type in Pantaneiro ewes also selected metatarsus circumference as the root node. The model indicated that 92% of animals had metatarsus circumferences below 15 cm; although most were classified as having a narrow muzzle, 27 individuals were misclassified as broad. Morphometric measurements of the shin allowed the formation of a tree with good discriminatory ability, yielding a predictive accuracy of 84.8%.

**Fig. 3:**
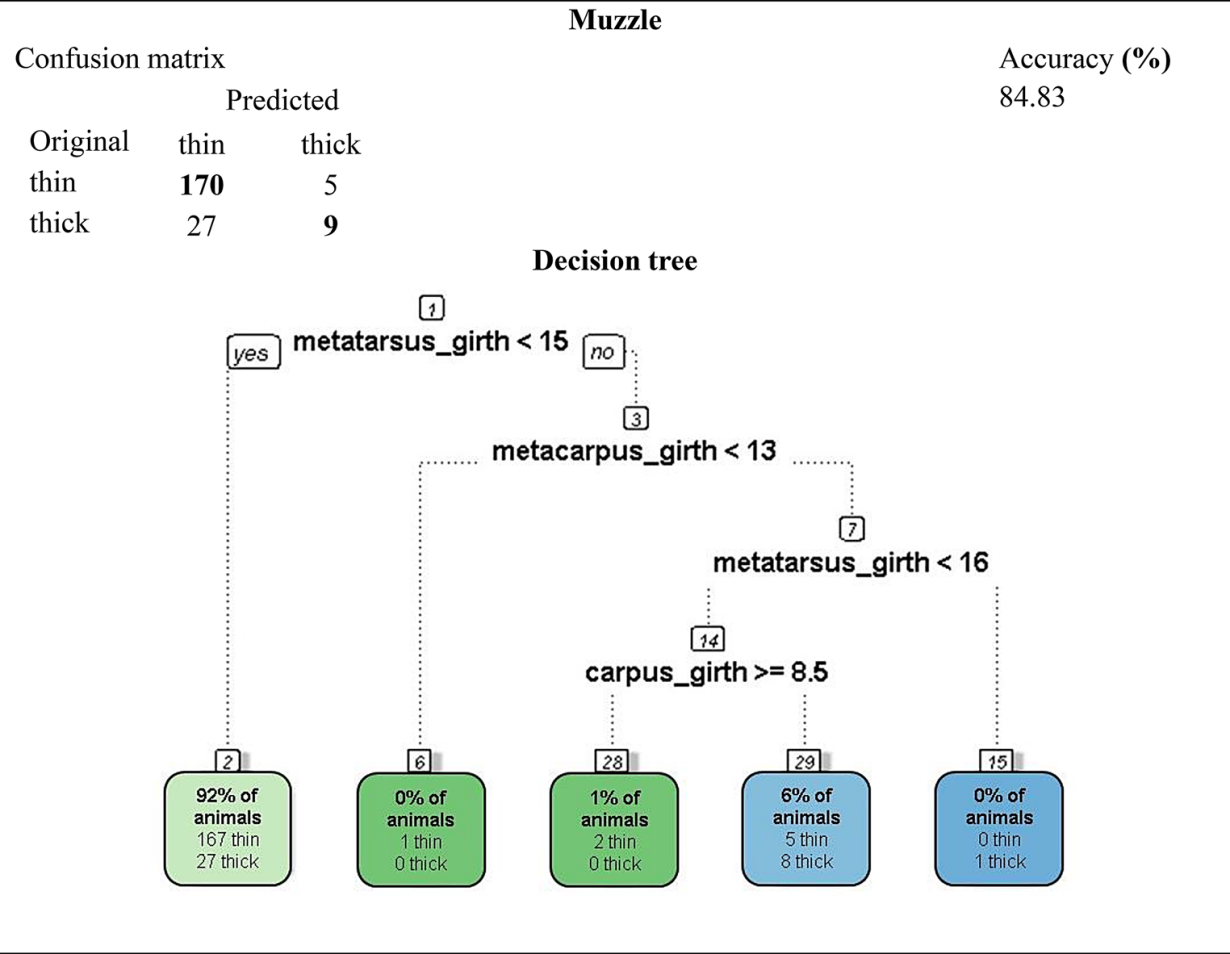
Decision tree and corresponding confusion matrix for predicting muzzle type (narrow vs. broad) in Pantaneiro ewes based on leg morphometric measurements

The corresponding confusion matrix (Fig. 3) shows that 170 ewes with a narrow muzzle and nine with a broad muzzle were correctly classified, confirming the predictive potential of distal limb dimensions for cranial phenotypes.

**Fig. 4:**
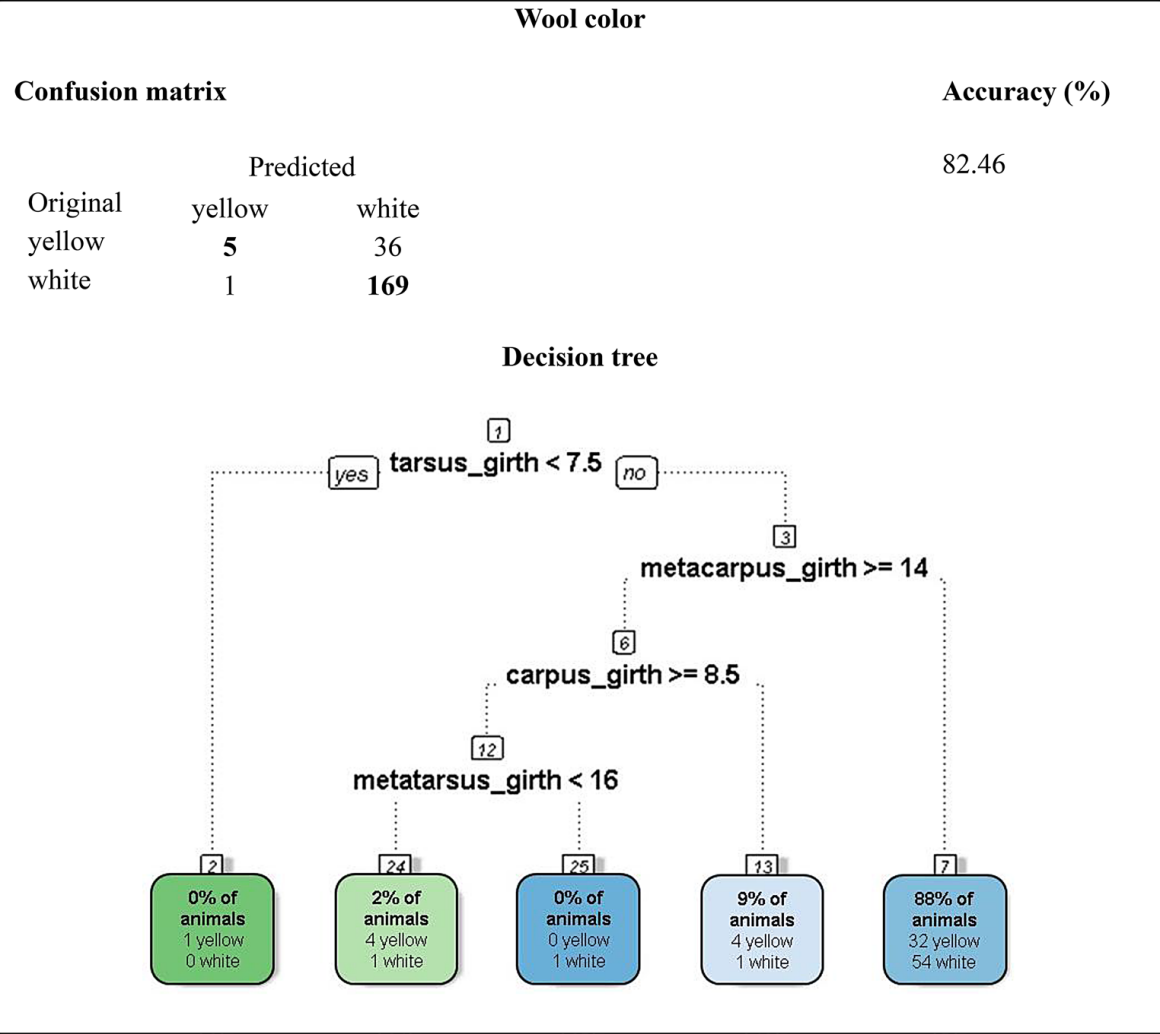
Decision tree for predicting wool color (white vs. yellow) in Pantaneiro ewes based on limb morphometric measurements

The model predicting wool color achieved an accuracy of 82.5% (Fig. 4). Metatarsus circumference was again selected as the root variable, with tarsus and metacarpus circumferences contributing to subsequent splits. Ewes with metatarsus circumference ≥ 13.5 cm and tarsus circumference ≥ 8.5 cm were predominantly classified as having yellowish wool (78%), whereas individuals below these thresholds tended to display white wool.

This result suggests that distal limb robustness may be phenotypically associated with pigmentation traits, possibly reflecting adaptive responses to solar radiation or thermoregulatory differences.

The seven remaining decision trees—representing traits such as presence of wool on the head, skin color, and body markings—achieved accuracies between 54.5% and 71.6% and are presented as Supplementary Figures (S1–S7). Although these models exhibited weaker predictive power, they provide complementary insights into the morphological plasticity of the Pantaneira breed and were included for transparency and reproducibility.

## Discussion

High coefficients of variation for body weight and body condition score (37.85% and 24.65%, respectively; Table 1) indicate that the studied flock consisted of ewes of different ages and physiological stages. This heterogeneity is typical of conservation and smallholder herds, where females in different productive and reproductive phases coexist, a common feature in low-input tropical systems extensively (Fao, 2015; McManus et al. 2010).

Pantaneiro ewes in the present population showed wide, tall, and deep thoracic measurements, as well as a large thoracic circumference, confirming, under current semi-extensive management conditions, the robust thoracic pattern historically described for the breed. Rather than redefining breed characteristics, these results quantitatively support and update previous descriptions, indicating the persistence of a well-developed thoracic cavity associated with digestive and cardiorespiratory capacity, traits linked to physical vigor and environmental adaptability (Yakubu 2010). Similar structural patterns were previously reported by Oliveira et al. (2014), who described a robust and proportionally balanced body conformation in this breed.

Factor analysis (Table 2) revealed relevant interactions among quantitative morphological traits, supporting phenotypic characterization and selection processes within the breed. Factor 1 (Body) grouped variables associated with cardiorespiratory capacity and harmonic trunk development. These characteristics reflect adequate metabolic and functional performance (Kunene et al. 2009), as simple biometric measurements, such as rump height, trunk length, and thoracic perimeter, correlate with body weight and carcass composition, reinforcing their importance as indirect indicators of productive potential and predictors of adaptive efficiency, according toBakhshalizadeh et al. (2016) andChay-Canul et al. (2024).

Factor 2 (Shank) comprised variables related to the robustness of the distal limbs, a trait directly linked to rusticity and mobility under grazing systems. A larger shank circumference favors denser bones and firmer musculature, improving locomotor efficiency and enabling animals to travel longer distances in search of forage and water, a fundamental characteristic of extensive production systems. Recent studies in sheep and goats demonstrate that bone and muscle morphology of the limbs is closely linked to locomotor performance and adaptability under pasture-based conditions (Güzel et al. 2025). In addition, feeding behavior profiles in grazing systems reinforce that displacement capacity directly influences access to resources and overall adaptability of animals in tropical and semi-arid environments (Dias e Silva and Abdalla Filho 2020). This robustness may also confer advantages during reproductive interactions, reinforcing the adaptive and functional value of these measurements, as supported by recent analyses integrating mobility patterns and behavioral monitoring of sheep under extensive systems (Hou et al. 2025).

Factor 3 (Height) included variables such as rump height, withers height, and fore- and hindlimb lengths, reflecting body linearity and vertical skeletal development. The positive association among these measurements indicates that taller sheep with longer limbs tend to exhibit elongated and agile body conformation, favoring locomotor efficiency and adaptation to extensive systems (Yang et al. 2016; Liu et al. 2024).

Factor 4 (Functional condition) was primarily defined by high loadings of body condition score, with additional contributions from live weight, shoulder width, and chest girth. In contrast to the other factors, which summarized stable skeletal and morphostructural dimensions, this factor represents variation related to the functional and nutritional status of the animals rather than to fixed body architecture. The prominence of BCS indicates that this axis captures short-term phenotypic variation influenced by feeding level, metabolic status, and physiological stage. The contribution of mass-related traits reflects their shared expression under changes in body reserves, without defining a distinct structural module. Thus, Factor 4 should be interpreted as a dynamic, management-sensitive dimension, complementary to the structural factors (Body, Shank, and Height), reinforcing the role of BCS as a functional indicator rather than a core descriptor of morphological conformation in Pantaneiro ewes.

More recent evidence reinforces this perspective. Selvan et al. (2023) demonstrated in Malaimadu native cattle from India that multivariate morphometric analysis effectively explains body structural organization and reveals adaptive patterns in tropical ecosystems. Similarly, Chebo et al. (2024) quantified phenotypic variability in indigenous Ethiopian chickens using morphometric measurements, highlighting the relevance of body modularity for sustainable production systems in rural environments. Together, these findings emphasize the value of multivariate morphometric analysis as a robust tool for genetic improvement and sustainable management of locally adapted breeds, confirming phenotypic modularity as a key mechanism underlying adaptation.

Although previous studies have not directly applied decision tree algorithms to sheep, the literature indicates that combining multivariate approaches (e.g., principal component analysis, discriminant analysis, and PLS-DA) with classification algorithms improves the accuracy of phenotypic pattern and predictor identification (Nunes et al. 2012). In this context, the use of decision tree models in the present study enabled interpretable exploration of the relationships between quantitative morphometric traits and qualitative phenotypic characteristics. Among the ten models tested (Table 3), three achieved accuracies above 80%, highlighting both the robustness of the models and the biological relevance of the identified relationships.

The tree for head profile (Fig. 2) achieved an accuracy of 86.3%, with metatarsal circumference as the central variable, suggesting a correlation between appendicular skeleton and cranial morphology. The muzzle type prediction model (84.8%; Fig. 3) showed that distal limb measurements influence craniofacial refinement, while the tree for wool color (82.5%; Fig. 4) revealed an association between coat pigmentation and body measurements, possibly reflecting adaptive effects or co-selection of phenotypic traits. The remaining trees, with accuracies ranging from 54.5% to 71.6%, are presented in the supplementary material due to lower precision and informational redundancy.

Overall, the results indicate that shank, rump, withers, and trunk measurements are consistent predictors of phenotypic variation in Pantaneiro sheep. Integrating multivariate morphometric analysis with decision tree modeling provides solid evidence for the identification of adaptative morphological markers, supporting sustainable breeding and resilience-based conservation strategies for this native genetic resource in the tropical Pantanal ecosystem.

## Conclusion

The integration of multivariate morphometric analysis and decision tree models proved to be effective in identifying structural patterns and phenotypic predictors in Pantaneiro sheep. Multivariate morphometric analysis combined with decision tree models effectively distinguished structural and functional components of phenotypic variation in Pantaneiro ewes. Body (F1), Shank (F2), and Height (F3) represented stable morphostructural dimensions associated with adaptation to extensive conditions, whereas Functional Condition (F4) reflected dynamic variation driven mainly by body condition score and live weight, indicating short-term nutritional and physiological status rather than fixed conformation. Trees with accuracies above 80% indicated that simple measurements, such as metatarsal and tarsal circumferences, can reliably predict cranial and tegumentary characteristics. These findings provide a basis for standardized phenotyping protocols and reinforce the adaptive and productive value of the breed, consolidating it as a strategic genetic resource for sustainable production systems.

## Supplementary Information

Below is the link to the electronic supplementary material.

Supplementary Material 1

Supplementary Material 2

Supplementary Material 3

Supplementary Material 4

Supplementary Material 5

Supplementary Material 6

Supplementary Material 7

## Data Availability

The raw data supporting the conclusions of this article will be made available by the authors on request.
