# Supplementary material for: Morphometric characterization and decision tree–based prediction of phenotypic traits in Pantaneiro sheep
Source: Trop Anim Health Prod. 2026 May 28;58(5):297. doi: 10.1007/s11250-026-05088-5 (PMC13219216; doi:10.1007/s11250-026-05088-5)
Supplement: Supplementary file 2 — Supplementary Material 2 [file 11250_2026_5088_MOESM2_ESM.docx]

| **Skin color** | |
| --- | --- |
| **Confusion matrix** | **Accuracy (%)** |
| \|  \| Predicted \| \| \| \| \| --- \| --- \| --- \| --- \| --- \| \| Original \| white \| despigmented \| dark \| blemished \| \| white \| **58** \| 33 \| 0 \| 0 \| \| despigmented \| 44 \| **57** \| 0 \| 0 \| \| dark \| 9 \| 5 \| **0** \| 0 \| \| blemished \| 3 \| 2 \| 0 \| **0** \| \|  \|  \|  \|  \|  \| | 54.50 |
| **Decision tree** | |
| 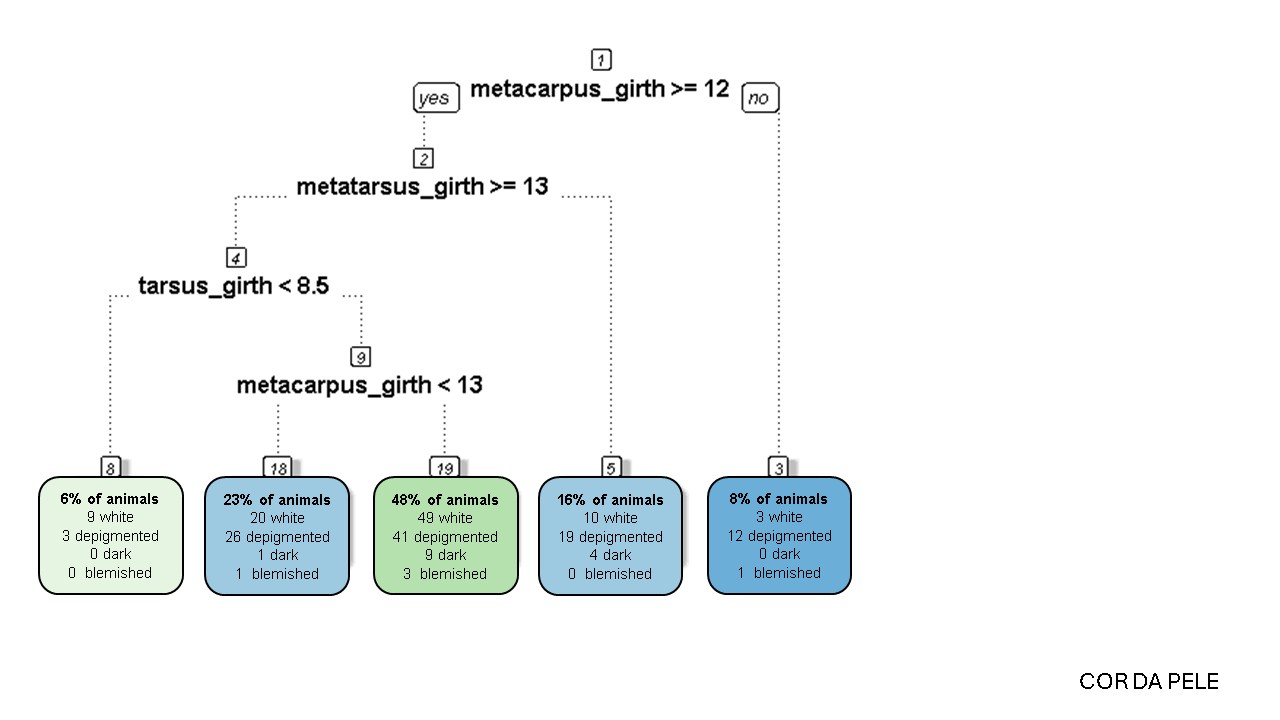 | |

**Supplementary Material S2.** Confusion matrix, classification accuracy, and decision tree generated using shin measurements of Pantaneiro sheep to predict skin color.
