# Supplementary material for: Morphometric characterization and decision tree–based prediction of phenotypic traits in Pantaneiro sheep
Source: Trop Anim Health Prod. 2026 May 28;58(5):297. doi: 10.1007/s11250-026-05088-5 (PMC13219216; doi:10.1007/s11250-026-05088-5)
Supplement: Supplementary file 4 — Supplementary Material 4 [file 11250_2026_5088_MOESM4_ESM.docx]

| **Head spots** | |
| --- | --- |
| **Confusion matrix** | **Accuracy (%)** |
| \|  \| Predicted \| \| \| --- \| --- \| --- \| \| Original \| present \| absent \| \| present \| **46** \| 44 \| \| absent \| 25 \| **96** \| \|  \|  \|  \| | 67.29 |
| **Decision tree** | |
| 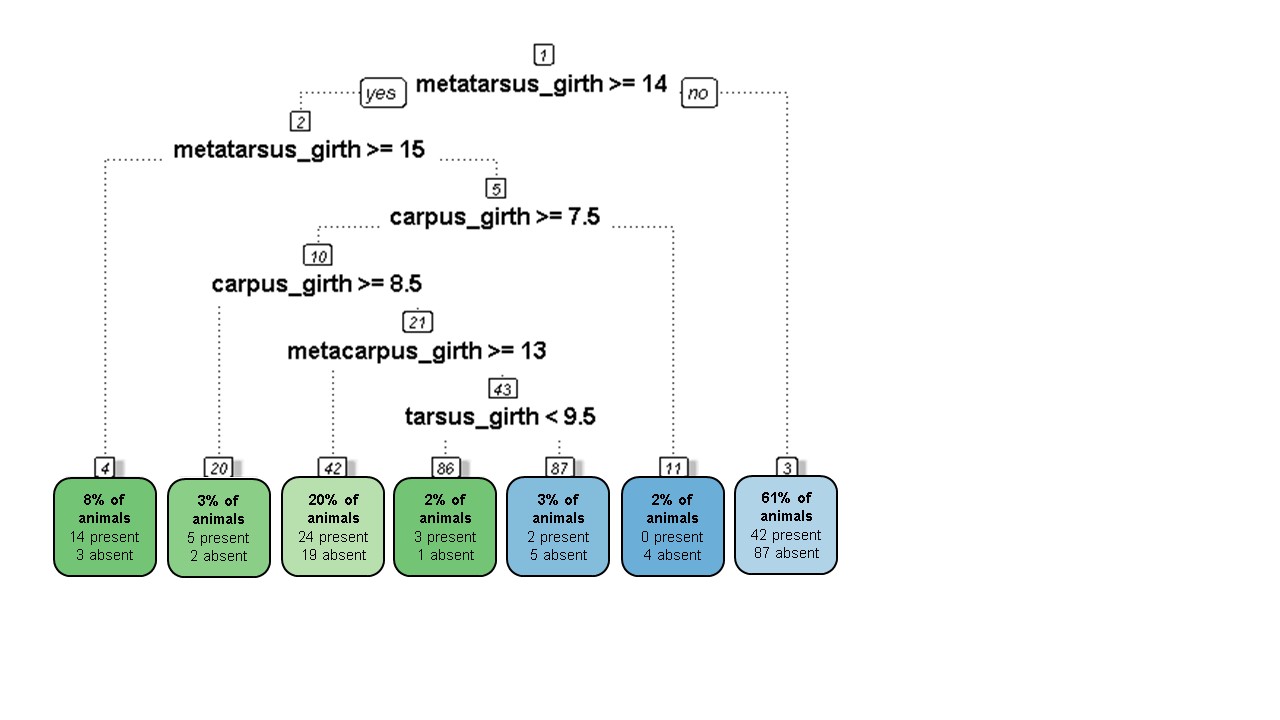 | |

**Supplementary Material S4.** Confusion matrix, classification accuracy, and decision tree generated using shin measurements of Pantaneiro sheep to predict the presence of head spots.
